# Taxon-Dependent Community Assembly of Bacteria and Protists in River Ecosystems: A Case Study from the Yujiang River

**DOI:** 10.3390/microorganisms13071650

**Published:** 2025-07-12

**Authors:** Yusen Li, Wenjian Chen, Yaoquan Han, Jianjun Lei, Bo Huang, Youjie Qin, Feng Lin, Caijin Li, Dapeng Wang, Lei Zhou

**Affiliations:** 1 Key Laboratory of Aquaculture Genetic and Breeding and Healthy Aquaculture of Guangxi, Guangxi Academy of Fishery Sciences, Nanning 530021, China; liyusen2019f@foxmail.com (Y.L.); wj.ch@foxmail.com (W.C.); hyqao208@foxmail.com (Y.H.); leijj206@foxmail.com (J.L.); hb800209@foxmail.com (B.H.); suyang371084562@foxmail.com (Y.Q.); linfeng0409@foxmail.com (F.L.); 2 China (Guangxi)-ASEAN Key Laboratory of Comprehensive Exploitation and Utilization of Aquatic Germplasm Resources, Ministry of Agriculture and Rural Affairs, Guangxi Academy of Fishery Sciences, Nanning 530021, China; 3 Aquatic Livestock and Veterinary Technology Promotion Station of Hengzhou City, Nanning 530300, China; licai374010918@foxmail.com; 4 University Joint Laboratory of Guangdong Province, Hong Kong and Macao Region on Marine Bioresource Conservation and Exploitation, College of Marine Sciences, South China Agricultural University, Guangzhou 510642, China

**Keywords:** environmental DNA, microbial diversity, species turnover, community assembly mechanisms, river ecosystem

## Abstract

Understanding the processes that drive microbial community assembly is a fundamental question in ecology, with important implications for predicting community responses to environmental disturbances. River ecosystems are under growing pressure from human disturbances, jeopardizing their ecological functions. Here, we investigated bacterial and protistan communities along the Yujiang River using environmental DNA metabarcoding. Bacterial communities exhibited significantly greater alpha diversity and broader habitat niches compared to protists. Additionally, a negative correlation was found between alpha diversity and niche breadth for both groups. Protistan communities exhibited significantly higher beta diversity (Bray–Curtis distance) than bacterial communities, with species turnover being the principal factor driving the variations in both communities. Null model results indicated that heterogeneous selection primarily structured bacterial communities, while stochastic processes (drift) mainly governed protist communities. Redundancy analysis and Mantel tests showed significant associations between environmental factors (e.g., temperature and pH) and bacterial community composition. Moreover, the longitude of sampling sites was linked to spatial variations in both bacterial and protistan communities. Further analyses, including distance-decay patterns, variation partitioning, and multiple regression on distance matrices, demonstrated that bacterial communities were driven by both environmental and spatial factors, while protist communities exhibited a stronger response to spatial factors. These results enhance our understanding of microbial community assembly in river ecosystems and provide valuable insights for the conservation and sustainable management of freshwater systems.

## 1. Introduction

River ecosystems are known to harbor complex microbial communities, dominated by bacteria and protists. These microorganisms are considered fundamental drivers of biogeochemical processes and ecosystem functioning [1]. River bacterial communities are of fundamental importance to river biomes and ecosystem structure, playing essential roles in material transformation, energy flow, and maintaining the self-purification ability of river water environments [2]. Protists, most notably heterotrophic nanoflagellates and smaller ciliates, have been observed to play a pivotal role in regulating and shaping the diversity and activity of prokaryotic communities through a process known as grazing [3]. The influence of microbial diversity on ecosystem functioning has been demonstrated in the scientific literature [4], with the body of research indicating that greater microbial diversity tends to enhance ecosystem multifunctionality and temporal stability [5]. Alpha diversity in river ecosystems is defined as the species richness and evenness within local microbial communities at specific sites or habitats [6]. Beta diversity is defined as the dissimilarity in microbial community composition between different sites, times, or environmental conditions [7]. In river ecosystems, patterns of beta diversity are indicative of both spatial and temporal variations in community structure, thereby providing insights into the processes that govern microbial biogeography [8]. It is imperative to comprehend the patterns and mechanisms that underpin microbial diversity in these dynamic environments, as this is essential for predicting ecosystem responses to environmental changes and for the effective management of aquatic resources [9].

The assembly of microbial communities is governed by a complex interplay of ecological processes such as selection, dispersal, ecological drift, and diversification [10]. These processes operate in a concurrent manner, yet their relative importance is subject to variation depending on environmental conditions, spatial scale, and microbial habitat characteristics [11]. Among them, environmental selection acts as a deterministic filter, where local abiotic conditions such as pH, temperature, salinity, and nutrient availability shape community composition by favoring taxa adapted to specific environmental conditions [12]. Conversely, the microbial community structures observed in rivers demonstrate rapid alterations along water flow paths, with bacterial and protist communities exhibiting a progressive downstream migration from their respective origins [13]. These alterations are indicative of the combined impact of elevated water turbulence, fluctuations in physicochemical conditions, and the relocation of microorganisms [14]. The relative importance of these factors is highly context-dependent, varying with ecosystem type, spatial scale, and microbial functional group [15,16,17]. Therefore, an understanding of the relative contributions of environmental filtering and geographic constraints is crucial for obtaining insights into the formation, persistence, and response of microbial communities to environmental change.

The utilization of eDNA metabarcoding has emerged as a rapid and non-invasive approach for biodiversity assessment and environmental monitoring, offering comprehensive insights into microbial dynamics within freshwater ecosystems [18]. In this study, eDNA metabarcoding was employed to investigate bacterial and protistan communities in the Yujiang River in China. Specifically, this study aimed to compare the diversity patterns of bacterial and protistan communities along the river and to further elucidate the driving factors shaping their variations.

## 2. Materials and Methods

### 2.1. Study Area and Sample Collection

**Figure 1:**
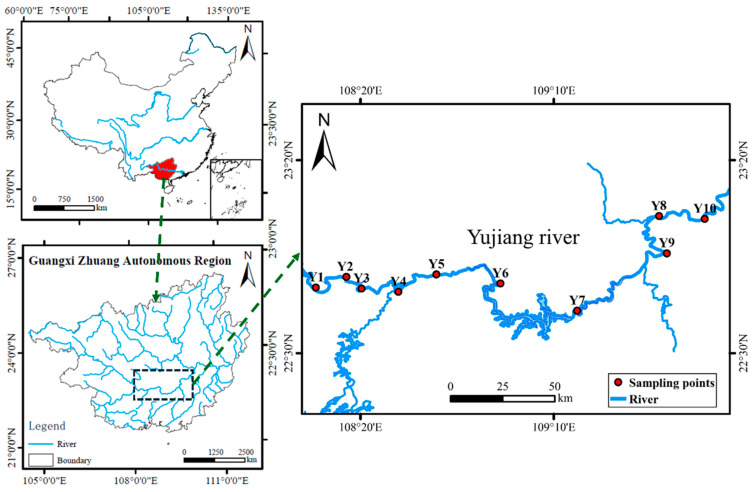
Map of sampling site.

The Yujiang River is the largest tributary of the Xijiang River, which is part of the Pearl River system in southern China. As a typical riverine ecosystem in this region, the Yujiang River is subject to both natural and anthropogenic influences. These characteristics make the Yujiang River a representative model for investigating microbial ecology in subtropical freshwater ecosystems. Water samples were collected from 10 sites along the Yujiang River in October 2023 (Figure 1). At each station, approximately 2 L of water was collected. Firstly, 1 L of water was immediately filtered through 0.22-micrometer polycarbonate membranes. The filter membranes were subjected to quick-freezing in liquid nitrogen, following which they were stored at −80 °C until DNA extraction. A further 1000 mL of water samples was stored temporarily at 4 °C for the purpose of conducting a series of environmental variable analyses.

### 2.2. Measurements of Environmental Factors

Water temperature, dissolved oxygen, specific conductance, total dissolved solids, pH, oxidation–reduction potential, ammonia nitrogen, and nitrate nitrogen were measured in situ using YSI ProPlus (YSI Inc., Yellow Springs, OH, USA). Water transparency was determined using a Secchi disk. Total phosphorus was measured using the spectrophotometric method, following the national standard method GB 11893-89 [19].

### 2.3. DNA Extraction and High-Throughput Sequencing

Genomic DNA was extracted from the water samples retained on the filters using the DNeasy PowerWater Kit (QIAGEN, Germantown, MD, USA), following the manufacturer’s protocol. The success of DNA extraction was verified by 1% agarose gel electrophoresis. DNA concentration and purity were assessed using a NanoDrop ND-1000 Spectrophotometer (NanoDrop Technologies, Wilmington, DE, USA). To characterize the microbial communities, the V4 region of the eukaryotic 18S rRNA gene and the 16S rRNA gene were amplified to target protists and bacteria, respectively. The primer pairs used were TAReuk454FWD1F: CCAGCASCYGCGGTAATTCC and TAReukREV3R: ACTTTCGTTCTTGATYRA for the 18S rRNA gene [20], and 515FmodF: GTGYCAGCMGCCGCGGTA and 806RmodR: GGACTACNVGGGTWTCTAAT for the 16S rRNA gene [21]. The resulting PCR products were purified and sequenced on the Illumina PE250 platform (Illumina Inc., San Diego, CA, USA) according to the standard sequencing procedures. The amplicon data was uploaded to the NCBI SRA (PRJNA1277653).

### 2.4. Data Processing

Amplicon sequencing data were processed following established workflows from previous studies [22,23]. In brief, paired-end reads were merged, quality-filtered, and dereplicated using the --fastq_mergepairs, --fastx_filter, and --derep_fulllength commands in VSEARCH v2.28.1 [24]. Non-redundant sequences were subsequently denoised into amplicon sequence variants (ASVs) using the --unoise3 algorithm in USEARCH [25]. Taxonomic classification of ASVs was performed with the SINTAX algorithm in USEARCH. Protistan ASVs (from the 18S rRNA gene) were annotated using the PR2 v5.1.0 database [26], while bacterial ASVs (from the 16S rRNA gene) were classified against the SILVA v138 database [27].

### 2.5. Statistical Analysis

All statistical analyses were carried out in R (v4.3.0) [28]. The alpha diversity (Chao1 index) was calculated using the vegan package v2.7-1 [29]. Each ASV’s niche breadth was assessed based on Levin’s index using the MicroNiche package v1.0.0 [30]. Community-level habitat niche breadth was defined as the average niche breadth of all ASVs detected in a given sample [31]. Furthermore, the bacterial and protistan ASVs were classified as generalists or specialists based on the niche breadth index, with 3 and 1.5 being the thresholds used for this classification [32].

To disentangle the contributions of species replacement and richness difference to community turnover, beta diversity (Bray–Curtis dissimilarity) was partitioned using the agricolae package v1.3-7 and adespatial package v0.3-28 and visualized with the ggtern package v3.5.0 and vcd package v1.4-13 [33]. Community assembly processes were further explored using a whole-community null model based on the beta nearest taxon index (βNTI) and the Raup–Crick metric (RC) [34]. Deterministic processes were identified when |βNTI| > 2, with βNTI > 2 indicating heterogeneous selection and βNTI < −2 indicating homogeneous selection. Stochastic processes were inferred when |βNTI| ≤ 2, with further classification into homogeneous dispersal (RC < −0.95), dispersal limitation (RC > 0.95), or ecological drift (|RC| ≤ 0.95). Differences in beta diversity and βNTI between bacterial and protistan communities were also tested using the Wilcoxon rank-sum test.

Redundancy analysis (RDA) was applied to identify key environmental and geographic drivers of beta diversity using the vegan package v2.7-1. Mantel tests, performed via the LinkET package v0.0.3 [35], were used to assess correlations between beta diversity and environmental or geographic distances. Environmental dissimilarity among samples was calculated based on Euclidean distance, while geographic distances between sampling stations were computed using the geosphere package v1.5-20 [36] based on latitude and longitude coordinates. Distance-decay relationships between community similarity and environmental or geographic distances were evaluated using linear regression. Finally, variation partitioning analysis (VPA) and multiple regression on distance matrices (MRM) were conducted to quantify the relative contributions of environmental and spatial factors to shaping microbial beta diversity, using the vegan package v2.7-1 and ecodist package v2.1.3 [37], respectively.

## 3. Results

### 3.1. Differences in the Alpha Diversity and Niche Breadth Between Bacteria and Protists

**Figure 2:**
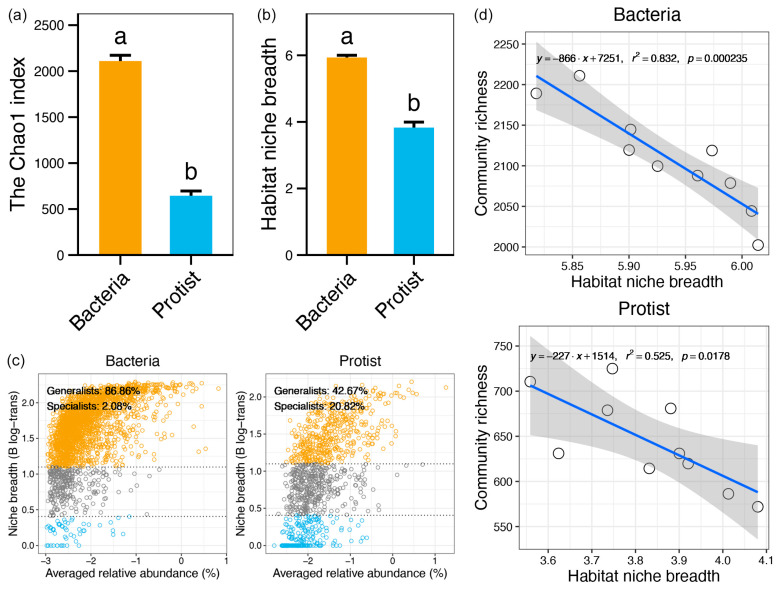
Comparisons of alpha diversity (**a**) and habitat niche breadth (**b**) between bacterial and protistan communities. (**c**) Distribution of specialists and generalists in bacterial versus protistan communities. (**d**) Linear regression of habitat niche breadth with their alpha diversity. The blue line in each sub-figure represents the fitted curve, and the gray shadow represents the 95% confidence interval. Different lowercase letters indicate significant differences between groups (*p* < 0.05).

The Chao1 index of bacterial communities ranged from 2000 to 2250, while that of protistan communities ranged from 550 to 750 along the Yujiang River. The Wilcoxon rank-sum test showed that the Chao1 index of bacterial communities was significantly higher than that of protistan communities in the Yujiang River (*p* < 0.05, Figure 2a). Similarly, bacterial communities showed a significantly broader habitat niche compared to protistan communities in the Yujiang River (*p* < 0.05, Figure 2b). Based on niche breadth classification, generalists accounted for 86.86% of bacterial communities, while specialists comprised only 2.08% (Figure 2c). In contrast, protistan communities had fewer generalists (42.67%) and more specialists (20.82%). In addition, a strong inverse relationship was identified between habitat niche breadth and species richness for both bacteria and protists (linear regression, *p* < 0.05), with a stronger relationship in bacteria (r^2^ = 0.832) than in protists (r^2^ = 0.525).

### 3.2. Assembly Mechanisms of Bacterial and Protistan Communities

**Figure 3:**
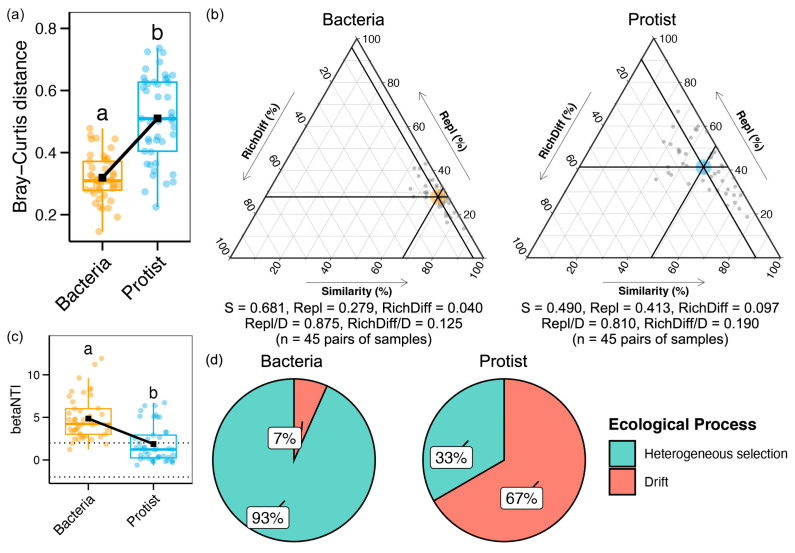
(**a**) Difference in the beta diversity between bacterial and protistan communities (Wilcoxon test, *p* < 0.05). (**b**) Comparison of species replacement and richness difference between bacterial and protistan communities. (**c**) Difference in the betaNTI between bacterial and protist communities. (**d**) Ecological processes in shaping the assembly of bacteria and protists. Different lowercase letters indicate significant differences between groups (*p* < 0.05).

The Bray–Curtis distance was calculated to assess community variation along the Yujiang River. Protistan communities exhibited significantly higher Bray–Curtis dissimilarity than bacterial communities (*p* < 0.05, Figure 3a), indicating greater spatial variation in protists. Beta-diversity decomposition showed that species replacement was the dominant contributor to community turnover in both groups (Figure 3b), with a slightly higher replacement observed in bacteria (0.875) compared to protists (0.810). Additionally, bacterial communities displayed significantly higher βNTI values than protistan communities (*p* < 0.05, Figure 3c), suggesting stronger deterministic assembly in bacteria, while protistan communities were more influenced by stochastic processes based on the median βNTI. Combined βNTI and RC analyses further suggested that the assembly of bacterial and protistan communities was shaped through a balance between heterogeneous selection and drift (Figure 3d).

### 3.3. Driving Factors for Variations in Bacterial and Protistan Communities

**Figure 4:**
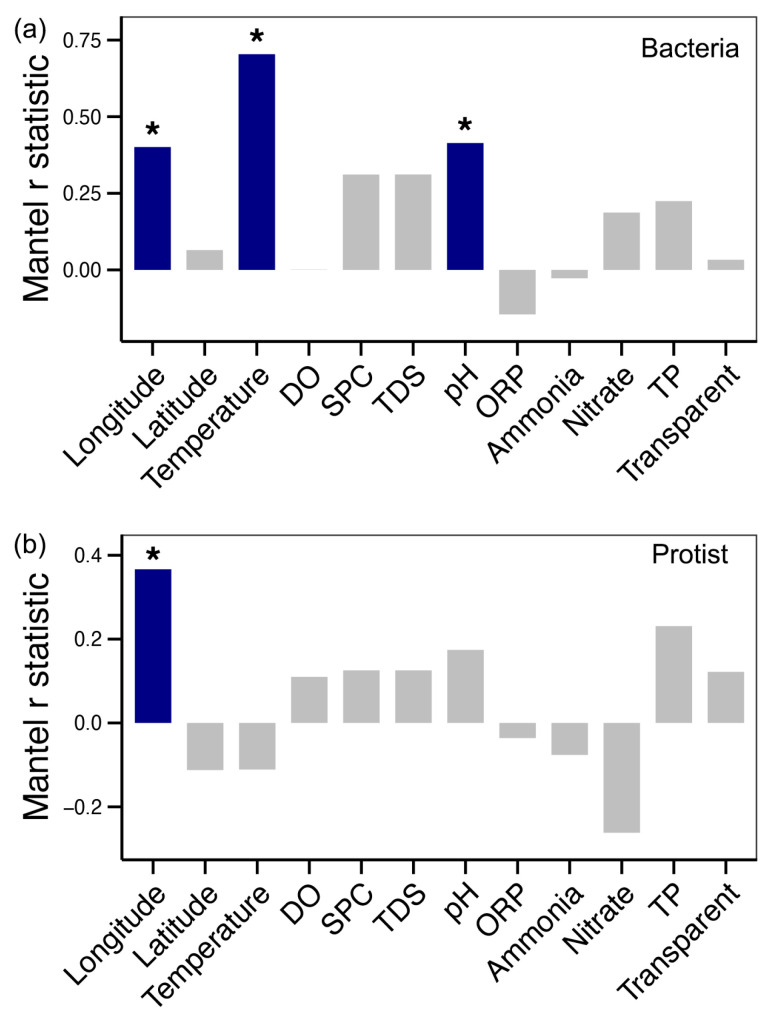
Mantel test results showing the correlations of bacterial (**a**) and protistan (**b**) communities with spatial and environmental factors along the Yujiang River. Significant correlations (*p* < 0.05) are highlighted by blue bars with asterisks.

RDA showed that bacterial community variation was significantly associated with water temperature, dissolved oxygen (DO), specific conductance (SPC), total dissolved solids (TDS), pH, and the longitude of sampling sites (*p* < 0.05, Table 1). In contrast, protistan community variation was less strongly correlated with longitude (*p* < 0.05, Table 1). Mantel test results further confirmed that longitude was associated with both bacterial and protistan compositions (*p* < 0.05, Figure 4). Additionally, bacterial communities showed strong correlations with water temperature and pH (*p* < 0.05, Figure 4a).

**Figure 5:**
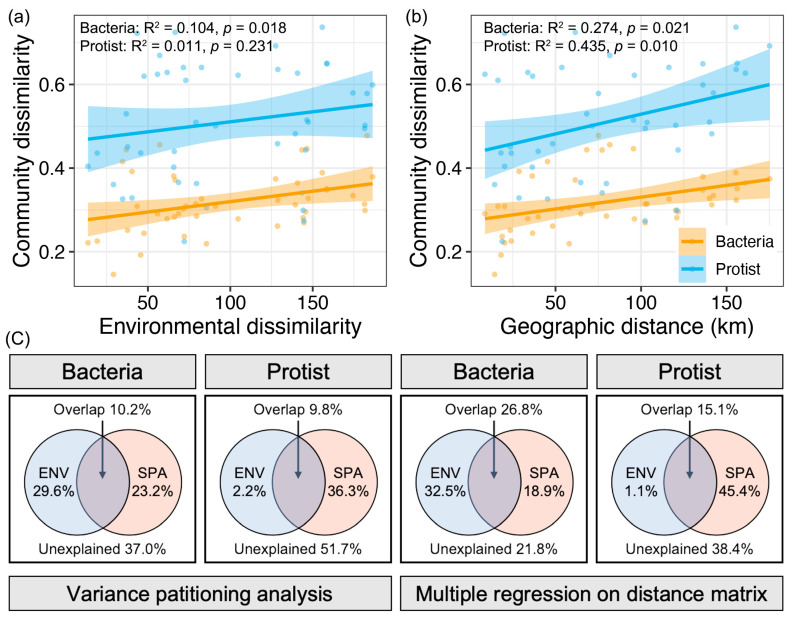
Relationships between community similarity (Bray–Curtis) and (**a**) environmental similarity (Euclidean distance) and (**b**) geographic distance for bacterial and protistan communities. The line in each sub-figure represents the fitted curve, and the gray shadow represents the 95% confidence interval. (**c**) VPA and MRM results for bacterial and protistan communities based on environmental and spatial variables.

A significant distance decay of community similarity based on environmental variables was observed in bacterial communities (*p* < 0.05), but not in protistan communities (*p* > 0.05, Figure 5a). In contrast, geographic distance significantly affected both bacterial and protistan communities (*p* < 0.05, Figure 5b). Variation partitioning analysis (VPA) and multiple regression on distance matrices (MRM) further quantified these effects. For bacterial communities, environmental and spatial factors contributed comparably to community variation (VPA: 29.6% vs. 23.2%; MRM: 32.5% vs. 18.9%), with environmental effects being slightly stronger (Figure 5c). In protistan communities, spatial effects overwhelmingly dominated (VPA: 36.3% vs. 2.2%; MRM: 45.4% vs. 1.1%) (Figure 5c). Together, these results indicate that bacterial community variations were jointly driven by environmental and spatial factors, whereas protistan community variations were primarily shaped by spatial processes.

## 4. Discussion

The bacterial communities in the Yujiang River exhibited greater species richness and broader environmental adaptability compared to protists (Figure 2). River ecosystems are typically characterized by high biodiversity, with studies reporting thousands of species-level taxa [38]. The higher abundance, metabolic versatility, and rapid reproduction rates of bacteria confer competitive advantages in both species richness and environmental tolerance within riverine systems [39]. In contrast, although protists are ecologically significant and contribute substantially to biodiversity, they tend to display heightened sensitivity to local environmental conditions and lower connectivity, which may restrict their overall richness and ecological breadth in comparison to bacteria [40]. These results suggest that bacterial communities may possess greater resistance and stability when subjected to external disturbances, such as anthropogenic activities, compared to protistan communities. Additionally, a negative correlation was observed between microbial richness and niche breadth along the Yujiang River (Figure 2d). Similar patterns have been widely reported in meta-analyses, where species-rich communities often exhibit narrower niche breadths, typically interpreted as evidence of increased ecological specialization [31,41]. This phenomenon may be explained by trade-offs among niche dimensions: specialization along one ecological axis often comes at the expense of performance along others, ultimately leading to narrower niche breadth in species-rich communities [42].

The balance between deterministic and stochastic processes is a fundamental determinant of microbial community assembly. As illustrated in Figure 3c, the bacterial communities in the Yujiang River were primarily structured by deterministic processes, while protistan communities were more strongly influenced by stochastic processes. Similar deterministic assembly patterns for bacteria have been reported in river systems, such as the Chishui River [43]. In contrast, stochasticity has been identified as the dominant driver shaping protistan communities, where dispersal limitation and random immigration play key roles [44]. Deterministic processes, driven by abiotic and biotic environmental selection, generate niche differentiation that actively filters bacterial community composition [45]. Protists, on the other hand, typically exhibit lower dispersal capacity relative to bacteria, and their assembly appears to be less tightly constrained by environmental filtering [9]. The dominance of stochastic processes in protists may be further influenced by river hydrodynamics, flow variability, and seasonal rainfall, which collectively shape protistan dispersal and colonization patterns [46]. These contrasting assembly mechanisms likely reflect fundamental differences in ecological traits and dispersal abilities between bacteria and protists in river ecosystems. Overall, bacterial communities in rivers are more strongly shaped by environmental selection, whereas protistan communities are more affected by random dispersal events and ecological drift [47].

It is imperative to comprehend the relative significance of environmental versus geographic factors, as this knowledge is of practical importance for the effective management and restoration of ecosystems [48]. Numerous studies have shown that environmental conditions typically exert a stronger influence on microbial community assembly than geographic distance alone [49,50]. For example, pH has been identified as a key environmental filter that regulates microbial survival and proliferation in specific habitats [51]. Additionally, increasing water temperature has been shown to promote phototrophic Cyanobacteria while reducing the abundance of chemoheterotrophic bacteria [52]. In this study, environmental variables such as temperature and pH emerged as the primary drivers of bacterial community variation along the Yujiang River (Figure 4a, Table 1). Moreover, bacterial communities appeared to be more sensitive to environmental fluctuations than protistan communities (Figure 5). In highly disturbed environments, deterministic processes are often amplified as environmental stress intensifies [53]. Consequently, microbial responses to future environmental changes are likely to be more closely driven by changing local conditions rather than by purely spatial factors. However, as environmental gradients shift, the geographic distribution of suitable habitats may also be altered, indirectly influencing microbial biogeography. This highlights the potential for bacterial communities to serve as more sensitive indicators of environmental change in river ecosystems compared to protists.

While environmental filtering is a key factor in microbial community assembly, geographic effects also play a substantial role, particularly when considering scale-dependent patterns [54]. In this study, geographic factors appeared to have a more pronounced influence on protistan community structure (Figure 5). The significance of spatial factors is known to vary with spatial scale and the dispersal capabilities of different taxa [55]. At local scales (from meters to a few kilometers), community assembly is usually dominated by environmental heterogeneity [56]. However, at broader regional scales (extending hundreds of kilometers), the influence of geographic distance becomes more evident. The sampling sites spanned several hundred kilometers along the Yujiang River, which may partly explain the pronounced influence of spatial factors observed in this study. It is well documented that microbial communities generally exhibit weaker distance-decay patterns compared to macroorganisms due to their high dispersal potential [57]. Nevertheless, despite being microorganisms, protists have substantially larger cell sizes than bacteria [58]. It has been hypothesized that this larger body size may constrain protistan dispersal, thereby enhancing the strength of distance-decay patterns in protistan communities relative to bacterial communities [59], a possibility that warrants further investigation. These findings suggest that the effective monitoring and management of microbial communities in river ecosystems may require differentiated strategies. Bacterial communities, which are more closely tied to local environmental conditions, can be effectively assessed and managed through targeted, site-specific interventions. In contrast, protistan communities, which exhibit stronger geographic structuring, may demand broader, spatially coordinated sampling and management efforts to account for spatial dispersal limitations. This highlights the importance of tailoring monitoring programs to the ecological characteristics of different microbial groups to ensure accurate assessment and effective ecosystem management.

## 5. Conclusions

This study elucidated the distinct assembly mechanisms of bacterial and protistan communities along the Yujiang River. Bacterial communities exhibited higher richness, broader niche breadth, and stronger environmental adaptability, suggesting greater resilience compared to protists. Both communities were primarily shaped by species turnover; however, bacteria were predominantly structured by environmental filtering, while protist communities were more influenced by geographic factors, likely due to their limited dispersal capacity. These findings highlight the need for microbial management strategies that account for the differing ecological drivers of bacteria and protists. Future studies should further explore the ecosystem functions and services provided by riverine microbial communities to better support aquatic ecosystem management.

## Figures and Tables

**Table 1:** RDA of the relationships between microbial communities and environmental factors.

	Bacteria		Protist	
	r^2^	*p*-Value	r^2^	*p*-Value
Temperature	0.876	**0.001**	0.064	0.756
DO	0.546	**0.048**	0.499	0.091
SPC	0.903	**0.002**	0.219	0.418
TDS	0.903	**0.002**	0.219	0.418
pH	0.943	**0.001**	0.363	0.188
ORP	0.030	0.894	0.347	0.231
Ammonia	0.260	0.352	0.243	0.405
Nitrate	0.423	0.156	0.069	0.718
TP	0.239	0.391	0.574	0.055
Transparent	0.273	0.315	0.413	0.167
Longitude	0.877	**0.002**	0.748	**0.027**
Latitude	0.549	0.066	0.287	0.280

## Data Availability

The data that support the findings of this study are openly available from the NCBI at https://www.ncbi.nlm.nih.gov/, reference number PRJNA1277653 [National Center for Biotechnology Information], accessed on 10 July 2025.
